# Regulation of Anthocyanin Biosynthesis by Drought and UV-B Radiation in Wild Tomato (*Solanum peruvianum*) Fruit

**DOI:** 10.3390/antiox11091639

**Published:** 2022-08-24

**Authors:** Gerardo Tapia, Monserrat Castro, Carlos Gaete-Eastman, Carlos R. Figueroa

**Affiliations:** 1 Unidad de Recursos Genéticos, Instituto de Investigaciones Agropecuarias, INIA Quilamapu, Chillán 3800062, Chile; 2 Faculty of Forest Sciences, Universidad de Concepción, Concepción 4070386, Chile; 3 Institute of Biological Sciences, Universidad de Talca, Talca 3465548, Chile; 4 Laboratory of Plant Molecular Physiology, Institute of Biological Sciences, Universidad de Talca, Talca 3465548, Chile; 5 Millennium Nucleus for the Development of Super Adaptable Plants (MN-SAP), Santiago 8340755, Chile

**Keywords:** anthocyanin, drought stress, R2R3 MYB transcription factor, *Solanum peruvianum*, UV radiation, wild tomato

## Abstract

Anthocyanins are plant pigments derived from the phenylpropanoid pathway which are produced in many different species, contributing to defense against stresses by their antioxidant properties. Cultivated tomatoes cannot synthesize flavonoids; however, wild tomatoes such as *Solanum chilense* and *Solanum lycopersicoides* have anthocyanin pigmented skin. Other wild tomato species such as *Solanum peruvianum* have been poorly studied concerning anthocyanin accumulation in the fruit. This research is the first to address the regulation of anthocyanin biosynthesis mediated by drought stress and light radiation in *S. peruvianum* fruit. Transcript accumulation of *SpAN2*, encoding for a key MYB type transcription factor for the regulation of anthocyanin biosynthesis, was induced in the fruit of plants exposed to drought treatment. In addition, fruit peel accumulates a greater anthocyanin content in water deficit-treated plants. The expression of *SpAN2* was also regulated according to sunlight exposure, reaching a higher expression during maximal daily UV radiation and under controlled UV-B treatments. Similar results were observed for the expression of the late flavonoid biosynthetic gene dihydroflavonol 4-reductase (*SpDFR*). These results suggest that *SpAN2* and *SpDFR* are involved in anthocyanin biosynthesis under drought stress and UV radiation in *S. peruvianum*.

## 1. Introduction

Anthocyanins are an important class of flavonoids that represent a large group of plant secondary metabolites. Anthocyanins are glycosylated polyphenolic compounds with a range of colors varying from orange, red, and purple to blue in flowers, seeds, fruits, and vegetative tissues [1]. Anthocyanins protect plants against various biotic and abiotic stresses like drought and UV radiation [2,3], partially due to their powerful antioxidant properties. In addition, anthocyanin-rich food products have become increasingly popular due to their attractive colors and suggested benefits for human health [4,5].

Most higher plants can synthesize anthocyanins, but the exact nature of the anthocyanins formed can differ widely in plant species due to the activity of specific enzymes that add sugars, methyl groups, and acyl residues to the basic anthocyanidin structure [6]. Anthocyanin biosynthesis is generally catalyzed by a total of nine conserved enzymes: phenylalanine ammonium-lyase (PAL), cinnamate 4-hydroxylase (C4H), *p*-coumaroyl 4-CoA ligase (4CL), chalcone synthase (CHS), chalcone isomerase (CHI), flavanone 3-hydroxylase (F3H), dihydroflavonol 4-reductase (DFR) and anthocyanidin synthase (ANS). The DFR enzyme marks the start of the specific pathway for anthocyanin formation [7,8] catalyzing the conversion of flavonols (myricetin, kaempferol, and quercetin) into anthocyanins. It is therefore considered a key enzyme in the formation of anthocyanins, in which case myricetin, which is a flavonol, would be the precursor of delphinidin. Studies with purple-colored transgenic tomatoes (*Solanum lycopersicum*) indicated that delphinidin together with petunidin is the type of anthocyanin found in the highest proportion in tomato fruit peel [9], delphinidin being the largest class of anthocyanins observed in tomatoes [10].

Anthocyanin production at the transcriptional level is regulated by the conserved regulatory complex MBW (MYB-bHLH-WD40), which is composed of three types of transcriptional regulators [11,12]. In plants, MYB transcription factors (TFs) are involved in controlling diverse metabolic pathways (including the anthocyanin pathway), development, signal transduction, and disease resistance [13]. The two-repeat R2R3 MYB protein is said to be directly associated with the regulation of anthocyanin synthesis. The R2R3 MYB proteins constitute one of the largest families of TFs in plants, with 146 R2R3 MYB genes identified in *Arabidopsis thaliana* [14]. Overexpression of the *AtPAP1* gene, (Production of Anthocyanin Pigment 1 or *AtMYB75*) results in anthocyanin accumulation in *Arabidopsis thaliana* [15]. Analysis of these TFs in apples (*Malus* × *domestica*) exposed to high light, UV radiation, and low temperatures showed an increase in the *MdMYB* transcript correlating with anthocyanin accumulation [16]. Other studies in *M.* × *domestica* and strawberry (*Fragaria* × *ananassa*) have shown a strong correlation between the *MYB10* gene and anthocyanin levels during fruit development [17,18]. However, many repressors in anthocyanin synthesis are also MYB-type TFs, including the strawberry MYB1 (FaMYB1) [19], and the recently reported SlMYBATV in tomato (*S. lycopersicum*) [20,21].

*Anthocyanin 2* (*SlAN2*) and *anthocyanin 1* (*SlANT1*), genes encoding for MYB-type TFs, have previously been identified and are suggested to be involved in anthocyanin synthesis in *S. lycopersicum* [22]. Three loci, *Anthocyanin fruit* (*Aft*), *atroviolaceae* (*atv*), and *Aubergine* (*Abg*), could increase anthocyanin production in *S. lycopersicum* when they are introgressed from wild tomatoes [23]. The *Aft* locus was originally identified in *Solanum chilense*, which causes anthocyanin accumulation in green immature fruit, through stimulation by high light [9]. The *atv* locus, which was originally identified from *Solanum cheesmaniae*, has been shown to influence anthocyanin pigmentation throughout the tomato plant, particularly in vegetative tissues [9]. Additionally, *Abg* locus was identified from *Solanum lycopersicoides*, showing a similar *Aft* phenotype, increasing anthocyanin accumulation under high solar radiation, but has a variegated expression [9]. Indeed, a study was conducted to evaluate the expression of these factors in tomato mutant plants expressing the *Aft* and *atv* loci, showing that both *SlAN2* and *SlANT1* were expressed in all genotypes that produced anthocyanins in the skin of the fruit [22]. A recent study demonstrates a new gene, *SlAN2-like*, belonging to the *Aft* locus, which is the key gene responsible for the *Aft* phenotype [23].

*Solanum peruvianum* is one of the wild relatives of the cultivated tomato and has been a source of many resistance genes, especially to drought, and could be a useful genetic resource for protection from UV radiation, considering recently published data that showed a relationship between UV-B exposure and anthocyanin production in cultivated tomato seedlings [24] and fruit [25]. These wild species show a strong purple coloring in the field, which is related to their ability to accumulate anthocyanins in their fruits [26].

Therefore, to start a characterization of the antioxidant potential in wild tomatoes, in the present research we analyzed the expression pattern of the key genes *DFR* and *AN2* for anthocyanin synthesis for the first time in the wild tomato *S. peruvianum* in response to solar and UV-B radiation and drought stress.

## 2. Materials and Methods

### 2.1. Plant Material and Growth Conditions

Seeds from the two *Solanum peruvianum* accessions QUI958 and QUI3954 were obtained from a collection maintained at the germplasm collection of the Genetic Resource Unit (GRU) at Agricultural Research Institute (INIA-Quilamapu), Chillán, Chile (latitude 36°34′ S; longitude 72°06′ W). Both accessions come from a natural area of the Tarapacá Region, Chile (latitude 18°14′ S; longitude 70°09′ W; elevation 355 m.a.s.l.). Plant growth conditions were according to Tapia et al. [27]. Briefly, seeds were germinated in pots containing a mixture of vermiculite and sand (5:1). Once developed, the seedlings were transferred to 20-L pots containing a mixture of soil, sand, and vermiculite (1:1:1) and fertilized using Basacote Plus 3M (COMPO, Münster, Germany). The plants were grown under greenhouse conditions with natural light until flowers were obtained, which were manually pollinated to obtain fruit.

### 2.2. Water and Light Stress-Related Treatments

Water treatments were performed for QUI958 and QUI3954 accessions during their reproductive development from flowering to ripe fruit stages (about three months) under greenhouse conditions. Three plants per accession for both optimal and restricted watering treatments were irrigated daily and three times per week maintaining a 60–65% and 20–22% of volumetric water content (VWC) in the soil, respectively. Soil water content (% VWC) was measured using ProCheck with sensor GS1 (Decagon Devices Inc., Pullman, WA, USA). For each accession and treatment, three replicates of a pool of 7–10 ripe fruit each (one per plant) were used for anthocyanin quantification and gene expression analyses.

To perform sunlight radiation experiments plants of QUI958 accession were grown in an open environment during summer at GRU. The ripe fruit were exposed to direct solar radiation during a diurnal period and three replicates of a pool of 7–10 fruit each (one per plant) were collected at three different times of the day (9:00, 12:00, and 16:00 h) and analyzed for gene expression. UV-B treatment was performed over tomato fruit growing under greenhouse conditions. Six plants with fruit at the green developmental stage attached to the plant were fixed to a platform to reach a uniform exposure and avoid shading. Fruit samples from three plants were harvested previously for UV-B treatment and used a 0 h-UV-B treatment. UV-B radiation was supplied by a UV tube at 312 nm (T8-M, Vilber Lourmat, Eberhardzell, Germany) for 24 h from midday, and harvested fruit of the remaining three plants were used as 24 h-UV-B treatment. Three replicates of a pool of 7–10 fruit each (one per plant) from 0 and 24 h UV-B treatments were utilized for gene expression analyses.

### 2.3. Anthocyanin Quantification

Anthocyanin determination was performed according to the methodology described by Giusti and Wrolstad [28]. Briefly, three biological replicates (three pools of ripe fruit) from both *S. peruvianum* accessions under water treatments were processed. Fruit peel was removed, and 0.2 g of tissue per replicate was weighed and stored at −80 °C. To perform anthocyanin extraction, the tissue was ground in a mortar with liquid nitrogen, and 1.5 mL of a mix of cold methanol, HCl, and water (90:1:1 *v*/*v*/*v*) was added to the ground tissue and centrifuged for 10 min at 9000 rpm at 4 °C. The supernatant was sonicated and centrifuged again. An aliquot of the sonicated product was used for spectrophotometric measurements at 540 nm and the content was expressed as milligrams of delphinidin-3-glucoside equivalent per 100 g of fruit peel.

### 2.4. Molecular Analyses

The RNA extraction kit ‘SV total RNA isolation system’ (Promega, Madison, WI, USA) was used to extract total RNA from the fruit peel of three biological replicates (three pools of fruit) according to the manufacturer’s instructions. RNA integrity was evaluated by denaturing gel electrophoresis and RNA concentration was determined spectrophotometrically (Epoch spectrophotometer, Biotek Instruments, Winooski, VT, USA). Then, 1 μg of purified total RNA was used for reverse transcription using the ‘Superscript III cDNA Synthesis’ kit (Invitrogen, Waltham, MA, USA) according to the manufacturer’s instructions. For the reverse transcription quantitative PCR (RT-qPCR) experiment, samples were amplified by using the ‘SensiMix SYBR HI-ROX’ kit (Meridian bioscience, Memphis, TN, USA) in ECO real-time PCR equipment (Illumina). PCR conditions were as follows: 95 °C for 10 min, and 40 subsequent cycles of 95 °C for 15 s, 60 °C for 15 s, and 72 °C for 15 s, with a melting curve from 55 °C to 90 °C at 0.5 °C increments. *EF1-alpha* and *UBI-E4* genes were used as the internal reference genes to standardize cDNA concentration. Relative gene expression was calculated using the method described by Pfaffl [29]. Table S1 shows the specific primer sequences for genes. Sequences of experimental genes were obtained from *Solanum peruvianum* transcriptome reported by our group recently [30] and deposited in the NCBI GenBank database (Accession Nos. ON568202 and ON568201 for *SpDFR* and *SpAN2*, respectively). Sequences from normalizer genes were cross amplified from *S. lycopersicum* orthologous genes from GenBank NCBI (https://www.ncbi.nlm.nih.gov/genbank/, accessed on 23 May 2022, Table S1).

### 2.5. Statistical Analysis

Results were expressed as mean ± standard deviation (SD) from triplicate measurements. Statistical analysis was performed by first testing data normality by using the Shapiro–Wilk test, and then using analysis of variance (ANOVA) followed by Duncan’s multiple comparison test performed for total anthocyanin content and expression analysis of *SpAN2* under drought stress. For expression analysis of *SpDFR* under drought stress, a non-parametric analysis of variance was performed using the Kruskal–Wallis’s test, because this data set failed the normality distribution test. One-way ANOVA followed by Tukey’s multiple comparison test and mean differences at *p* < 0.05 were considered significant for expression analysis of *SpAN2* and *SpDFR* under sunlight radiation. Finally, because only the UV-B factor was tested, the unpaired Student’s *t*-test for expression analysis of *SpAN2* and *SpDFR* under UV-B radiation was performed. All statistical analyses were performed by using GraphPad Prism version 9.4.0 for macOS (GraphPad Software, San Diego, CA, USA.

## 3. Results

### 3.1. Determination of Anthocyanin Content in Solanum Peruvianum Fruit under Drought Stress

**Figure 1:**
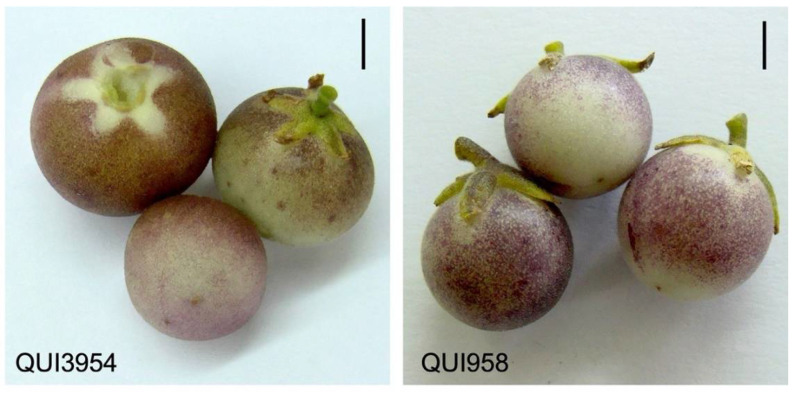
Representative ripe fruit of the *Solanum peruvianum* accessions QUI3954 and QUI958 of the germplasm collection of the Genetic Resource Unit (GRU) at Agricultural Research Institute (INIA-Quilamapu), Chillán, Chile. The bar in each panel represents 1 cm.

The ripe fruit of *Solanum peruvianum* accessions QUI3954 and QUI958 showed similar patterns of purple coloration on their peel according to the direct incidence of light, as can be seen in the areas surrounding the skin shaded by the calyx (Figure 1).

**Figure 2:**
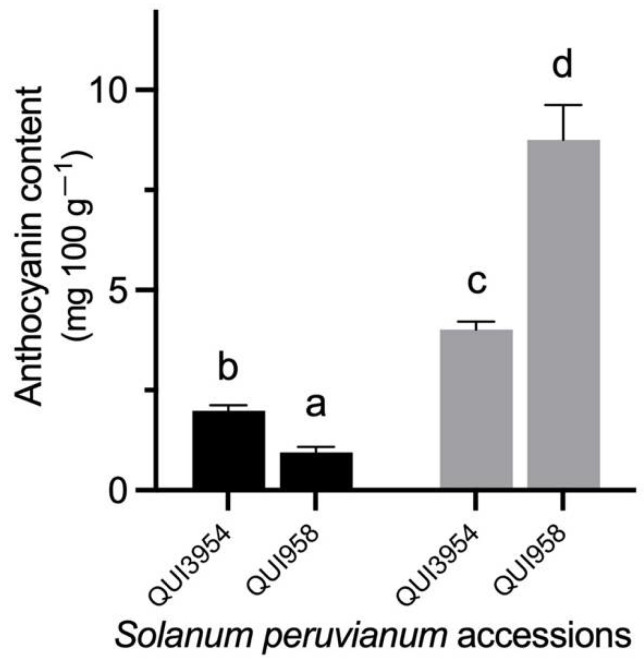
Anthocyanin content in the fruit peel of *Solanum peruvianum* QUI3954 and QUI958 accessions under drought stress. Samples from optimal- and restricted-watering plants are shown in black and grey bars, respectively. Content is expressed as mg of delphinidin 3-glucoside equivalent per 100 g of fruit peel. Error bars in each column indicate the standard deviation of three replicates. A Duncan’s multiple comparisons test was performed with 95% confidence. Different letters indicate significant differences between means. For details see the Section 2.

The QUI3954 and QUI958 accessions were selected for drought stress treatments and then the fruit was analyzed for anthocyanin content and gene expression analyses. An increase in the anthocyanin content was observed in both *S. peruvianum* accessions under drought stress (Figure 2). On average, QUI3954 showed a higher anthocyanin content in control conditions than QUI958, although the latter exhibited a remarkably increment of the anthocyanin content under drought stress.

### 3.2. Expression Analysis of SpAN2 and SpDFR in S. peruvianum Fruit under Drought Stress

**Figure 3:**
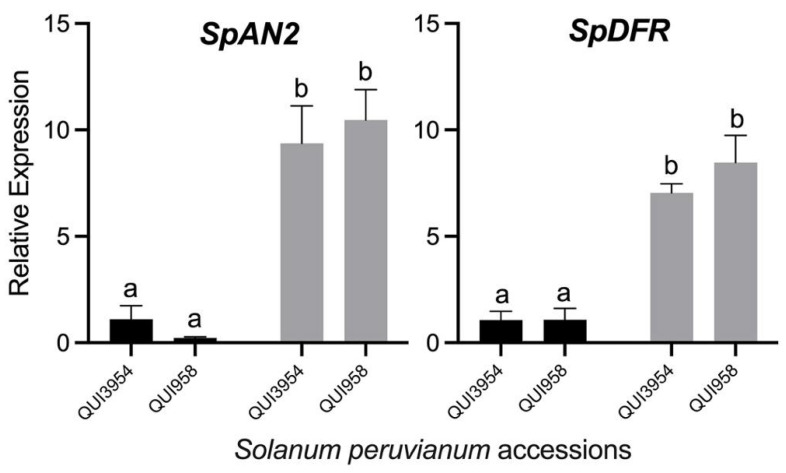
Relative expression levels of *SpAN2* and *SpDFR* genes in the fruit peel of *Solanum peruvianum* QUI3954 and QUI958 accessions under drought stress. Samples from well irrigated- and restricted watered plants are shown in black and grey bars, respectively. Error bars in each column represent the standard deviation of three replicates. For *SpAN2*, Duncan’s multiple comparisons test was performed with 95% confidence. In *SpDFR*, a non-parametric analysis of variance was performed using the Kruskal–Wallis test. Different letters indicate significant differences between means. For details see the Section 2.

To know the effect of drought on the expression of genes related to anthocyanin biosynthesis, we analyzed the transcript accumulation of key genes involved in anthocyanin biosynthesis in tomato fruit. Firstly, the *SpDFR* gene, which encodes for the enzyme that catalyzes the conversion of flavonols into anthocyanins, shows a significant increase in accumulation of its transcripts in both accessions under drought stress (Figure 3). Secondly, the gene encoding for the transcription factor *SpAN2* shows a similar expression pattern to that of *SpDFR*, showing an about 10-fold increment of transcript accumulation with respect to control samples (Figure 3).

### 3.3. Expression Analysis of SpAN2 and SpDFR in S. peruvianum Plants under Total Sunlight and UV-B Radiation

**Figure 4:**
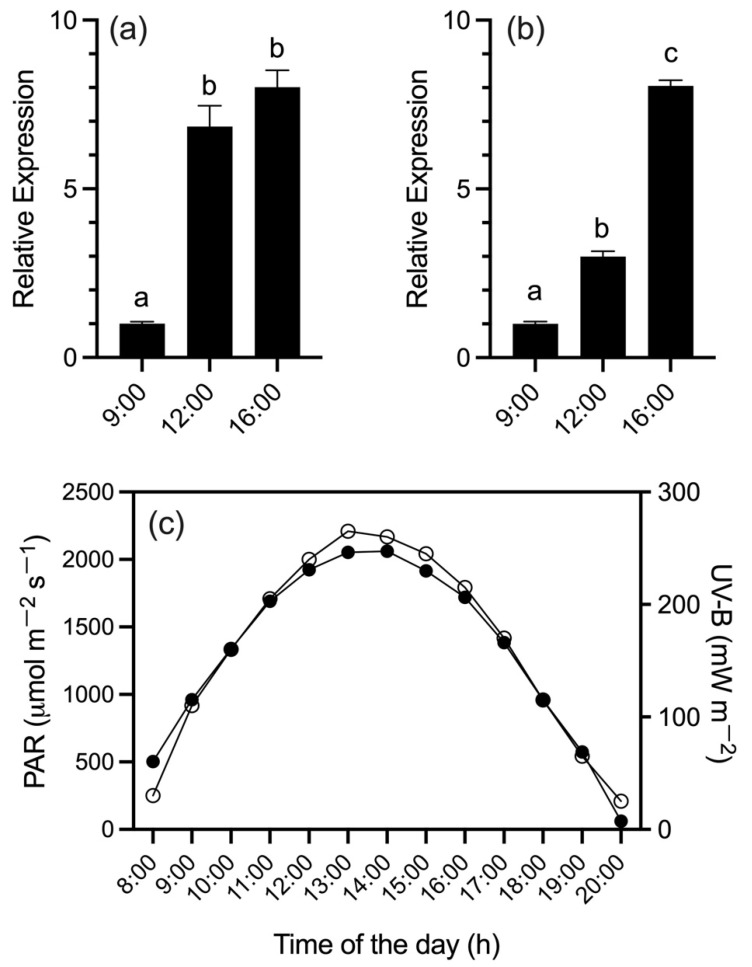
Relative expression levels of (**a**) *SpAN2* and (**b**) *SpDFR* genes in the fruit peel of *Solanum peruvianum* accession QUI958 under sunlight radiation. The expression of each gene was measured at 9:00, 12:00, and 16:00 h. Error bars in each column represent the standard deviation of three replicates. For both genes, Tukey’s multiple comparisons test was performed with 95% confidence. Different letters indicate significant differences between means. (**c**) The radiation index for UV-B (µW m^−2^) (white circles) and PAR (µmol m^−2^ s^−1^) (black circles) for the fruit sampling day (15 January 2020) is shown. For details see the Section 2.

Additionally, due to its higher accumulation of anthocyanin under drought stress, the QUI958 accession was selected to observe the transcript accumulation of *SpDFR* and *SpAN2* in fruit peel under total sunlight and UB-V radiation. First, transcript accumulation was evaluated three times during the day (9:00, 12:00, and 16:00 h). The results showed basal transcript accumulation levels at 9:00 h for the two genes under study (Figure 4A,B). Interestingly, between 9:00 and 12:00 h increased levels of transcript accumulation were observed for both genes. However, *SpAN2* shows the faster increment, with up to an eight-fold increment of transcript accumulation regarding basal levels at 9:00 h (Figure 4A). We observed that a similar transcript accumulation level was reached by *SpAN2* with four hours less radiation than *SpDFR,* indicating a previous activation of the regulatory-related genes for the light-induced flavonoid biosynthesis. In the place of the experiment, the photosynthetically active radiation (PAR) increased during the day, as well as UV-B radiation (Figure 4C), being directly proportional to the transcript accumulation of the genes of interest.

**Figure 5:**
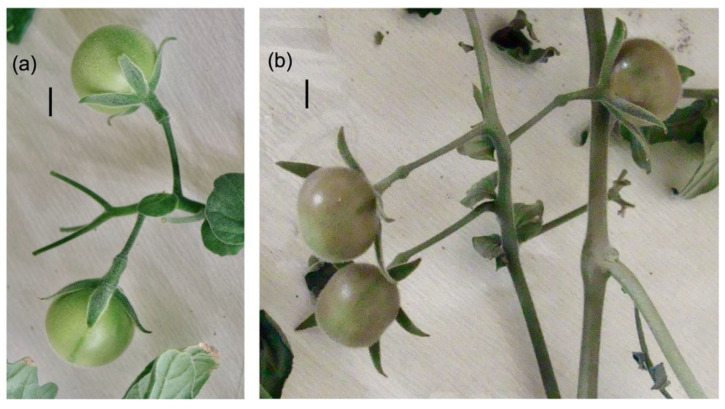
Fruit attached to *Solanum peruvianum* accession QUI958 plants of the UV-B experiment. UV-B-treated fruit at 0 and 24 h are shown in panels (**a**,**b**), respectively. The bar in each panel represents 1 cm. For details see the Section 2.

Considering that the specific effect of UV-B radiation on transcript accumulation cannot be determined under solar radiation, then an experiment under controlled conditions of UV-B treatment in greenhouse conditions was performed (Figure 5). In this case, *S. peruvianum* plants were exposed to 24 h of UV-B radiation, with the fruit peel showing a clear purple coloration (Figure 5).

**Figure 6:**
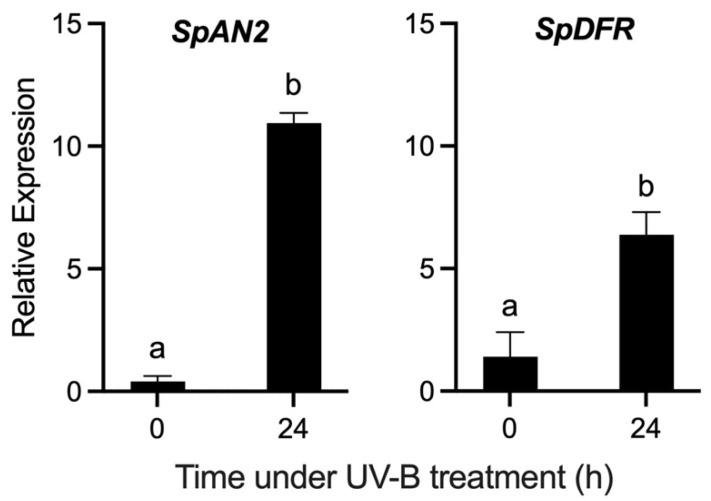
Relative expression levels of *SpAN2* and *SpDFR* genes in the fruit peel of *Solanum peruvianum* accession QUI958 under UV-B radiation treatment. The expression of each gene was measured at 0 and 24 h of UV-B radiation treatment. Error bars in each column represent the standard deviation of three replicates. The unpaired Student’s *t*-test was used to compare the means. Different letters indicate significant differences between means. For details see the Section 2.

A significant transcript accumulation for both genes was noticed up to 24 h after treatment (Figure 6). In particular, *SpAN2* shows the highest levels of transcript accumulation at the end of the experiment (Figure 6).

## 4. Discussion

*Solanum peruvianum* is a wild tomato species that is biogeographic and widely distributed from the South of Perú to the North of Chile, where it inhabits the Atacama Desert which is known for its extreme aridity [27,31]. Additionally, *S. peruvianum* produces small green mature fruit that accumulate anthocyanin pigment on their peel [31]. Until now, there is no evidence that anthocyanin content could change in response to some abiotic stress in *S. peruvianum* fruit. Our results suggest an increased accumulation of anthocyanin content in response to drought stress (Figure 1 and Figure 2). This phenomenon was previously seen in *Vitis vinifera* [32], where it was observed that water deficit led to an increase in methoxylated and hydroxylated anthocyanin derivatives, which would determine the specific pigment type and thus influence the coloring of the fruit peel. Additionally, the relationship between drought stress and anthocyanin content is well known, mainly because anthocyanins are osmoregulators and in this sense, plants can induce anthocyanin biosynthesis to maintain water homeostasis [3]. On the other hand, the induction of drought-coupled anthocyanin production can be a response to drought-stress coupled with reactive oxygen species (ROS) signaling. This hypothesis was tested in Arabidopsis and the identified drought-induced anthocyanins were directly associated with oxidative and drought stress tolerance [33]. This could be linked to the typical accumulation of anthocyanins produced into vacuoles, which are near sites of active ROS production, such as chloroplast and peroxisomes [3].

To get insight at the molecular level into the relationship between drought stress and anthocyanin accumulation in *S. peruvianum* fruit, two anthocyanin biosynthesis-related genes were studied. Firstly, *SpDFR*, which encodes for the enzyme dihydroflavonol 4-reductase and catalyzes the stereospecific reaction that converts dihydroflavonols to leucoanthocyanidins using NADPH as a cofactor, showed an increase in transcript accumulation under drought stress. This behavior has been reported in other anthocyanin-accumulating tomatoes, such as the introgressed *Solanum lycopersicum* ‘Ailsa Craig’ [22,34]. Finally, *SpAN2,* encoding for an MYB-type transcription factor (TF) that modulates the expression of the *DFR* gene and other genes in *Petunia hybrida* and ‘Ailsa Craig’ tomato [34,35], showed an increase in transcript accumulation under drought stress. Recent data suggest that four R2R3-MYB-type TFs regulate anthocyanin accumulation in tomato: SlAN2/SlMYB75, SlANT1, SlANT1-like, and SlAN2-like/Aft [22,34,36]. Recent studies have confirmed, by using functional approaches, that *SlAN2-like/Aft* regulates anthocyanin content in fruit tissue [21,23,37], and *SlAN2/SlMYB75* is a positive regulator for anthocyanin biosynthesis in vegetative tissues of *S. lycopersicum* ‘Indigo Rose’ [38]. Interestingly, in the Indigo Rose cultivar, both TFs are expressed in fruit, and there is no evidence which one could be responsible for anthocyanin accumulation under drought stress. Our data support the role of *SpAN2* as a positive regulator of anthocyanin accumulation under drought stress in *Solanum peruvianum*, which corresponds to a natural anthocyanin mechanism and is not because of an artificial introgression procedure, given all the evidence already known about anthocyanin production in tomato species.

Considering the biogeographic distribution of *S. peruvianum* [39], this wild tomato species is a good candidate to get an insight into solar radiation and UV-B exposure. Adding to our understanding of the effect on transcript accumulation of *SpDFR* and *SpAN2* under sunlight radiation, the peel of *S. peruvianum* fruit under this treatment showed increased expression levels for these two genes, suggesting that sunlight radiation has an impact on anthocyanin production, as a response to ROS production [40]. In *Arabidopsis,* both anthocyanin TF- and biosynthetic-related genes were induced under ROS production, where the anthocyanin content was increased [41]. Recent data showed in anthocyanin-accumulating tomato (Aft tomato) a significant anthocyanin accumulation in sunlight and blue + UV-B radiation treated fruit [25], which is in agreement with our data. To observe the specific effect of UV-B light on anthocyanin accumulation, plants of *S. peruvianum* were exposed to UV-B light. The obtained results showed the same transcript accumulation profile in fruit compared to the previous experiment, confirming the role of UV-B light stimulating anthocyanin biosynthesis in *S. peruvianum* fruit.

## 5. Conclusions

In the present research, we concluded that the fruit peel of *Solanum peruvianum* fruit has increased anthocyanin content under drought treatments and this correlates with the significant boost in the relative expression of *SpAN2* and *SpDFR* genes. Moreover, *SpAN2*, the gene encoding for the key MYB transcription factor that regulates the gene expression related to key enzymes for anthocyanin biosynthesis such as DFR, is highly regulated by UV-B light in the fruit peel of *S. peruvianum*. The diversity of anthocyanin composition and the transcriptional dynamics of the phenylpropanoid pathway-related genes in response to different abiotic stresses need to be more deeply investigated in *S. peruvianum* fruit. Finally, these results pave the way to deepen the regulation of anthocyanin biosynthesis in plant species that are exposed to extreme environmental conditions such as wild tomatoes and make *S. peruvianum* an interesting genetic resource for cultivated tomato breeding programs.

## Data Availability

Data is contained within the article and Supplementary Material.

## Supplementary Materials

The following are available online at https://www.mdpi.com/article/10.3390/antiox11091639/s1, Table S1: List of primers used in RT-qPCR analysis.

## References

[1] Tanaka Y., Sasaki N., Ohmiya A. (2008). Biosynthesis of Plant Pigments: Anthocyanins, Betalains and Carotenoids. Plant J..

[2] Del Valle J.C., Buide M.L., Whittall J.B., Valladares F., Narbona E. (2020). UV Radiation Increases Phenolic Compound Protection but Decreases Reproduction in Silene Littorea. PLoS ONE.

[3] Naing A.H., Kim C.K. (2021). Abiotic Stress-Induced Anthocyanins in Plants: Their Role in Tolerance to Abiotic Stresses. Physiol. Plant..

[4] Kay C.D., Pereira-Caro G., Ludwig I.A., Clifford M.N., Crozier A. (2017). Anthocyanins and Flavanones Are More Bioavailable than Previously Perceived: A Review of Recent Evidence. Annu. Rev. Food Sci. Technol..

[5] Mannino G., Gentile C., Ertani A., Serio G., Bertea C.M. (2021). Anthocyanins: Biosynthesis, Distribution, Ecological Role, and Use of Biostimulants to Increase Their Content in Plant Foods—A Review. Agriculture.

[6] Martin C., Zhang Y., Tomlinson L., Kallam K., Luo J., Jones J.D.G., Granell A., Orzaez D., Butelli E. (2012). Colouring up Plant Biotechnology. Recent Adv. Polyphen. Res..

[7] Kim B.G., Kim J.H., Kim J., Lee C., Ahn J.H. (2008). Accumulation of Flavonols in Response to Ultraviolet-B Irradiation in Soybean Is Related to Induction of Flavanone 3-β-Hydroxylase and Flavonol Synthase. Mol. Cells.

[8] Albert N.W., Lewis D.H., Zhang H., Irving L.J., Jameson P.E., Davies K.M. (2009). Light-Induced Vegetative Anthocyanin Pigmentation in Petunia. J. Exp. Bot..

[9] Mes P.J., Boches P., Myers J.R., Durst R. (2008). Characterization of Tomatoes Expressing Anthocyanin in the Fruit. J. Am. Soc. Hortic. Sci..

[10] Bovy A., de Vos R., Kemper M., Schijlen E., Almenar Pertejo M., Muir S., Collins G., Robinson S., Verhoeyen M., Hughes S. (2002). High-Flavonol Tomatoes Resulting from the Heterologous Expression of the Maize Transcription Factor Genes LC and C1. Plant Cell.

[11] Koes R., Verweij W., Quattrocchio F. (2005). Flavonoids: A Colorful Model for the Regulation and Evolution of Biochemical Pathways. Trends Plant Sci..

[12] Ramsay N.A., Glover B.J. (2005). MYB–BHLH–WD40 Protein Complex and the Evolution of Cellular Diversity. Trends Plant Sci..

[13] Jiang C.K., Rao G.Y. (2020). Insights into the diversification and evolution of R2R3-MYB transcription factors in plants. Plant Physiol..

[14] Stracke R., Werber M., Weisshaar B. (2001). The R2R3-MYB Gene Family in Arabidopsis Thaliana. Curr. Opin. Plant Biol..

[15] Borevitz J.O., Xia Y., Blount J., Dixon R.A., Lamb C. (2000). Activation Tagging Identifies a Conserved MYB Regulator of Phenylpropanoid Biosynthesis. Plant Cell.

[16] Allan A.C., Hellens R.P., Laing W.A. (2008). MYB Transcription Factors That Colour Our Fruit. Trends Plant Sci..

[17] Espley R.V., Hellens R.P., Putterill J., Stevenson D.E., Kutty-Amma S., Allan A.C. (2007). Red Colouration in Apple Fruit Is Due to the Activity of the MYB Transcription Factor, MdMYB10. Plant J..

[18] Castillejo C., Waurich V., Wagner H., Ramos R., Oiza N., Muñoz P., Triviño J.C., Caruana J., Liu Z., Cobo N. (2020). Allelic variation of MYB10 is the major force controlling natural variation in skin and flesh color in strawberry (*Fragaria* spp.) fruit. Plant Cell.

[19] Aharoni A., De Vos C.H.R., Wein M., Sun Z., Greco R., Kroon A., Mol J.N.M., O’Connell A.P. (2001). The Strawberry FaMYB1 Transcription Factor Suppresses Anthocyanin and Flavonol Accumulation in Transgenic Tobacco. Plant J..

[20] Cao X., Qiu Z., Wang X., Van Giang T., Liu X., Wang J., Wang X., Gao J., Guo Y., Du Y. (2017). A Putative R3 MYB Repressor Is the Candidate Gene Underlying Atroviolacium, a Locus for Anthocyanin Pigmentation in Tomato Fruit. J. Exp. Bot..

[21] Colanero S., Tagliani A., Perata P., Gonzali S. (2019). Alternative Splicing in the Anthocyanin Fruit Gene Encoding an R2R3 MYB Transcription Factor Affects Anthocyanin Biosynthesis in Tomato Fruits. Plant Commun..

[22] Povero G., Gonzali S., Bassolino L., Mazzucato A., Perata P. (2011). Transcriptional Analysis in High-Anthocyanin Tomatoes Reveals Synergistic Effect of Aft and Atv Genes. J. Plant Physiol..

[23] Yan S., Chen N., Huang Z., Li D., Zhi J., Yu B., Liu X., Cao B., Qiu Z. (2020). Anthocyanin Fruit Encodes an R2R3-MYB Transcription Factor, SlAN2-like, Activating the Transcription of SlMYBATV to Fine-Tune Anthocyanin Content in Tomato Fruit. New Phytol..

[24] Liu X., Zhang Q., Yang G., Zhang C., Dong H., Liu Y., Yin R., Lin L. (2020). Pivotal Roles of Tomato Photoreceptor SlUVR8 in Seedling Development and UV-B Stress Tolerance. Biochem. Biophys. Res. Commun..

[25] Kim M.J., Kim P., Chen Y., Chen B., Yang J., Liu X., Kawabata S., Wang Y., Li Y. (2021). Blue and UV-B Light Synergistically Induce Anthocyanin Accumulation by Co-Activating Nitrate Reductase Gene Expression in Anthocyanin Fruit (*Aft*) Tomato. Plant Biol..

[26] Gonzali S., Mazzucato A., Perata P. (2009). Purple as a tomato: Towards high anthocyanin tomatoes. Trends Plant Sci..

[27] Tapia G., Méndez J., Inostroza L. (2016). Different Combinations of Morpho-Physiological Traits Are Responsible for Tolerance to Drought in Wild Tomatoes Solanum chilense and Solanum peruvianum. Plant Biol..

[28] Giusti M.M., Wrolstad R.E. (2001). Characterization and Measurement of Anthocyanins by UV-Visible Spectroscopy. Curr. Protoc. Food Anal. Chem..

[29] Pfaffl M.W. (2001). A New Mathematical Model for Relative Quantification in Real-Time RT-PCR. Nucleic Acids Res..

[30] Tapia G., González M., Burgos J., Vega M.V., Méndez J., Inostroza L. (2021). Early Transcriptional Responses in Solanum peruvianum and Solanum lycopersicum Account for Different Acclimation Processes during Water Scarcity Events. Sci. Rep..

[31] Nakazato T., Franklin R.A., Kirk B.C., Housworth E.A. (2012). Population Structure, Demographic History, and Evolutionary Patterns of a Green-Fruited Tomato, Solanum peruvianum (Solanaceae), Revealed by Spatial Genetics Analyses. Am. J. Bot..

[32] Castellarin S.D., Pfeiffer A., Sivilotti P., Degan M., Peterlunger E., Di Gaspero G. (2007). Transcriptional Regulation of Anthocyanin Biosynthesis in Ripening Fruits of Grapevine under Seasonal Water Deficit. Plant Cell Environ..

[33] Nakabayashi R., Yonekura-Sakakibara K., Urano K., Suzuki M., Yamada Y., Nishizawa T., Matsuda F., Kojima M., Sakakibara H., Shinozaki K. (2014). Enhancement of Oxidative and Drought Tolerance in Arabidopsis by Overaccumulation of Antioxidant Flavonoids. Plant J..

[34] Kiferle C., Fantini E., Bassolino L., Povero G., Spelt C., Buti S., Giuliano G., Quattrocchio F., Koes R., Perata P. (2015). Tomato R2R3-MYB Proteins SlANT1 and SlAN2: Same Protein Activity, Different Roles. PLoS ONE.

[35] Quattrocchio F., Wing J., van der Woude K., Souer E., de Vetten N., Mol J., Koes R. (1999). Molecular Analysis of the Anthocyanin2 Gene of Petunia and Its Role in the Evolution of Flower Color. Plant Cell.

[36] Dubos C., Stracke R., Grotewold E., Weisshaar B., Martin C., Lepiniec L. (2010). MYB Transcription Factors in Arabidopsis. Trends Plant Sci..

[37] Sun C., Deng L., Du M., Zhao J., Chen Q., Huang T., Jiang H., Li C.B., Li C. (2020). A Transcriptional Network Promotes Anthocyanin Biosynthesis in Tomato Flesh. Mol. Plant.

[38] Zhi J., Liu X., Li D., Huang Y., Yan S., Cao B., Qiu Z. (2020). CRISPR/Cas9-Mediated SlAN2 Mutants Reveal Various Regulatory Models of Anthocyanin Biosynthesis in Tomato Plant. Plant Cell Rep..

[39] Chetelat R.T., Pertuzé R.A., Faúndez L., Graham E.B., Jones C.M. (2009). Distribution, Ecology and Reproductive Biology of Wild Tomatoes and Related Nightshades from the Atacama Desert Region of Northern Chile. Euphytica.

[40] Isah T. (2019). Stress and Defense Responses in Plant Secondary Metabolites Production. Biol. Res..

[41] Xu Z., Mahmood K., Rothstein S.J. (2017). ROS Induces Anthocyanin Production Via Late Biosynthetic Genes and Anthocyanin Deficiency Confers the Hypersensitivity to ROS-Generating Stresses in Arabidopsis. Plant Cell Physiol..

