# Supplementary material for: Regulation of Anthocyanin Biosynthesis by Drought and UV-B Radiation in Wild Tomato (Solanum peruvianum) Fruit
Source: Antioxidants (Basel). 2022 Aug 24;11(9):1639. doi: 10.3390/antiox11091639 (PMC9495367; doi:10.3390/antiox11091639)
Supplement: Supplementary file 1 [file antioxidants-11-01639-s001.zip › antioxidants-1849250-supplementary.pdf]

Table S1: List of primers used for reverse transcription quantitative PCR (RT-qPCR) analyses.

Gene Name	Gene Description	GenBank accession number	Forward primer	Reverse primer
AN2	Anthocyanin 2	ON568201	TTCCAGGAAGGACAGCAAAC	AACGAGGACGAGAATGAGGA
DFR	Dihydroflavonol 4-reductase	ON568202	CAAGGCAGAGGGAAGATTCATTTG	GCACCATCTTAGCCACATCGTA
UBI-E4	Ubiquitin conjugation factor E4	XM_004232138.4	AAGCAATGGATGCTGAGGCT	GAAGGTGCCGTTGAATGACA
EF1-alpha	Elongation factor1-alpha	NM_001247106.2	TGCTTGCTTTCACCCTTGGT	CGATTTCATCATACCTAGCCTTGGA
